# Ensiling of wheat straw decreases the required temperature in hydrothermal pretreatment

**DOI:** 10.1186/1754-6834-6-116

**Published:** 2013-08-14

**Authors:** Morten Ambye-Jensen, Sune Tjalfe Thomsen, Zsófia Kádár, Anne S Meyer

**Affiliations:** 1 Center for BioProcess Engineering, Department of Chemical and Biochemical Engineering, Technical University of Denmark, DK-2800 Kgs. Lyngby, DTU, Denmark

**Keywords:** Silage, Ensiling, Combined pretreatment, Hydrothermal treatment, Wheat straw, Enzymatic hydrolysis

## Abstract

**Background:**

Ensiling is a well-known method for preserving green biomasses through anaerobic production of organic acids by lactic acid bacteria. In this study, wheat straw is subjected to ensiling in combination with hydrothermal treatment as a combined pretreatment method, taking advantage of the produced organic acids.

**Results:**

Ensiling for 4 weeks was accomplished in a vacuum bag system after addition of an inoculum of *Lactobacillus buchneri* and 7% w/w xylose to wheat straw biomass at 35% final dry matter. Both glucan and xylan were preserved, and the DM loss after ensiling was less than 0.5%. When comparing hydrothermally treated wheat straw (170, 180 and 190°C) with hydrothermally treated ensiled wheat straw (same temperatures), several positive effects of ensiling were revealed. Glucan was up-concentrated in the solid fraction and the solubilisation of hemicellulose was significantly increased.

Subsequent enzymatic hydrolysis of the solid fractions showed that ensiling significantly improved the effect of pretreatment, especially at the lower temperatures of 170 and 180°C.

The overall glucose yields after pretreatments of ensiled wheat straw were higher than for non-ensiled wheat straw hydrothermally treated at 190°C, namely 74-81% of the theoretical maximum glucose in the raw material, which was ~1.8 times better than the corresponding yields for the non-ensiled straw pretreated at 170 or 180°C. The highest overall conversion of combined glucose and xylose was achieved for ensiled wheat straw hydrothermally treated at 180°C, with overall glucose yield of 78% and overall conversion yield of xylose of 87%.

**Conclusions:**

Ensiling of wheat straw is shown to be an effective pre-step to hydrothermal treatment, and can give rise to a welcomed decrease of process temperature in hydrothermal treatments, thereby potentially having a positive effect on large scale pretreatment costs.

## Background

Lignocellulosic residues such as wheat straw (WS) are an attractive renewable resource for the production of fuel, feed and chemicals. Wheat is the most important crop in the EU with an annual average production of over 130 Mt grain [1] and around 200 Mt of straw residues (using a residue to product factor of 1.5 according to [2]). Replacement of conventional sugar or starch based feedstock with lignocellulosic agricultural residues, such as WS, for ethanol production is advantageous due to a more efficient use of the agricultural area. However, lignocellulosic residues require more advanced processing technologies. Lignocellulose consists of the polysaccharides cellulose and hemicellulose and the polyphenolic structure of lignin; together forming a rigid matrix structure in the secondary plant cell wall. This structure is naturally ‘engineered’ to resist degradation, thus creating great challenges in terms of biorefining. Physical and chemical pretreatments have been developed for lignocellulosic biomass in order to create accessibility for hydrolytic enzymes to hydrolyze the polysaccharides into readily fermentable sugars [3]. Bioethanol production from lignocellulosic residues has been the main driver for the technology development, and production is now on the verge of industrialization [4]. However the industry is facing huge difficulties in creating enough economic viability to engage in full scale production [5]. Pretreatment have been shown to cover up to 33% of the processing costs [6-9]. The pretreatment step is most often based on hydrothermal principles of high temperatures (170-220°C) in aqueous solution, and is the most energy intensive and expensive process step in the lignocellulose to ethanol process, due to the need of high temperature, pressure, and/or chemicals as well as specialized equipment. Examples of pretreatment methods are hydrothermal treatment (HTT), dilute acid treatment (using H_2_SO_4_), and ammonia fiber explosion. HTT has been widely studied for pretreatment of WS and other cellulosic biomasses, where it facilitates high yields of enzymatic cellulose conversion (70-90%) and its simple approach without additives makes it advantageous to upscale [5,8,10,11] In the current Inbicon demonstration plant in Kalundborg, Denmark [5] the straw is hydrated to a dry matter (DM) mass fraction of 35% before it is continuously fed to a pressurized pretreatment reactor operating at 180-200°C for a retention time of 10-20 min [5]. Considering the low feed-in DM for lignocellulosic bioethanol, dry biomass storage processing is no longer an advantage as compared to traditional combustion. Furthermore drying of biomass increases the biomass recalcitrance towards biological degradation [12]. Alternatively wet storage (<40%DM) can be applied using ensiling.

Ensiling is the well-known preservation method for forages, based on anaerobic fermentation by lactic acid bacteria (LAB) that produce organic acids, reduce pH, and prevent growth of yeasts, fungi and competing bacteria. Lignocellulosic residues including WS, do not have sufficient available sugars to facilitate the necessary lactic acid fermentation required for preservation at low DM. Organic acids can be added directly instead of LAB fermentation [13], lignocellulytic enzymes can be applied to release fermentable carbohydrates from the lignocellulose [6], or sugars can be added as substrate for LAB fermentation [14]. This study applies the latter of the three strategies. The species of LAB are usually separated into homo- and heterofermentative LAB based on their type of hexose fermentation. The homofermentative utilizes the Empden-Meyerhof-Parnas pathway and produces only lactic acid, while the heterofermentative utilizes the phosphoketolase pathway and produce lactic- and acetic acid, ethanol and carbon dioxide [15]. However when pentoses are used as fermentation substrate, then both types of LAB may produce both lactic- and acetic acid, see Eq. 1, but variation do occur [16,17].

(1) $$\begin{array}{ccccc} \mathrm{Pentose} & & \mathrm{Lacti}\;\mathrm{cacid} & & \mathrm{Aceti}\;\mathrm{cacid}\\ \mathrm{HOC}H_{2}{\left(\mathrm{CH}\left(\mathrm{OH}\right)\right)}_{3}\mathrm{CHO} & \to & CH_{3}\mathrm{CH}\left(\mathrm{OH}\right)\mathrm{COOH} & + & CH_{3}\mathrm{COOH} \end{array}$$

Ensiling has in the last 6 years gained increased focus as a method for combined storage and pretreatment in biorefinery applications [6,18-24]. Based on studies of grass ensiling for forage purposes [25], the effect of ensiling as pretreatment is known to be correlated to the produced organic acids that act primarily on hemicellulose.

Oleskowicz-Popiel *et al*. [26] combined ensiling with HTT (190°C, 10 min) on maize, clover grass, and whole crop rye, which all contain easily fermentable free sugars, however they were not able to prove a positive effect of the ensiling. Xu *et al*. [27] studied the effect of adding lactic- and/or acetic acid to the hydrothermal pretreatment of dry corn stover and found that addition of acetic acid performed better as a catalyst than lactic acid, and increased the ethanol yield in a subsequent simultaneous saccharification and fermentation from 78% to 87% of the theoretical yield [27].

The pretreatment factors of temperature, holding time and pH, are often combined to one factor expressing the severity of the pretreatment [28]. Reducing pH through ensiling will increase the severity factor of the pretreatment at same temperature and holding time, thus higher severity would result in higher sugar release. It has however been shown by Pedersen et al. [29] that the use of the one dimensional severity factor to predict sugar yields is not reliable, because lignocellulosic pretreatment is much too complex.

Based on the hypothesis that the acid produced during ensiling can assist pretreatment, the aim of this study is to investigate the effect of ensiling prior to HTT in order to decrease pretreatment temperature and thereby decrease energy consumption. The ensiling is facilitated by addition of xylose and a heterofermentative LAB inoculum, which will favor acetic acid production in the silage. The motivation for using xylose as silage fermentation substrate is the availability of cheap C5 sugars in internal biorefinery process streams such as C5 molasses condensed from a HTT liquid fraction.

## Results and discussion

### Ensiling wheat straw

Ensiling of WS successfully preserved the biomass, resulting in only 0.35% loss in total DM and produced both acetic and lactic acid which caused the pH to drop from 7.0 to 3.7 (Table 1). The addition of 7 (w/w)% xylose resulted in 2.8 (w/w)% acetic acid and 2.4 (w/w)% lactic acid weight base in relation to the initial WS DM before ensiling. Over 1% of the added xylose was recovered, thus preservation can be carried out with less addition of xylose. Following Eq. 1 and assuming xylose were the only substrate, it can be calculated that 6 (w/w)% of utilized xylose would yield 3.6 (w/w)% lactic acid and 2.4 (w/w)% acetic acid. This is presumably due to the inoculum of *Lactobacillus büchneri* which is capable of a secondary fermentation where lactic acid is converted to acetic acid, thus shifting the ratio between acetic- and lactic acid [30,31]. The motive to favor acetic acid to lactic acid is that it increases the effect of pretreatment [27].

**Table 1:** Dry matter loss and pH after 4 weeks ensiling; the most significant organic compounds in water extraction after ensiling

	
**DM loss (w/w)%**	0.35
**pH**	3.69
**Glucose**	0.06 ± 0.00
**Xylose**	1.27 ± 0.02
**Xylitol**	0.17 ± 0.00
**Lactic acid**	2.46 ± 0.09
**Acetic acid**	2.79 ± 0.08
**Propionic acid**	0.36 ± 0.01
**Total**	7.06

Total includes the mentioned organic compounds.

Production of propionic acid and xylitol (Table 1) is due to minor secondary fermentations, which are still occurring during the stable phase of the ensiling. These secondary reactions can be carried out by a variety of acid tolerant microorganisms such as LAB, *Clostridium*-, *Bacillus*- or *Propioni* bacteria. It is well documented that secondary fermentation often utilizes other carbon sources than sugars including fatty acids, alcohols and amino acids derived from plant proteins [16]. This complicates the mass balance when products become substrates, for example parts of the produced lactic acid has most likely been further metabolized into propionic acid.

The ensiled wheat straw (EWS) was also analyzed for butyric acid, since butyric acidusually is due to presence of *Clostridium* bacteria and is a common indicator of insufficient preservation. The amounts detected were however below 0.01 (w/w)%, showing efficient preservation.

It was not possible in this experimental setup to distinguish between leftover xylose and the xylose released from hemicellulose. Preliminary experiments have shown xylose release during WS ensiling (unpublished observation, M. Ambye-Jensen and S. T. Thomsen), but in amounts less than 0.1 (w/w)%. It is therefore assumed that the released xylose only counts for a negligible fraction compared to leftover xylose. No arabinose was found in the water extractions and only insignificant amounts of released glucose were detected (Table 1).

The DM loss during ensiling was very limited and measured to below 0.5%. This was due to a fast and effective preservation facilitated by the efficient laboratory vacuum ensiling, however, losses cannot be expected to be as low in large scale.

Evaporation of fatty acids needs to be considered when determining DM content of silage, which can be done by using of volatilization coefficients to determine the acids lost during DM-determination [32]. In this work volatilization coefficients and the quantity of the total fatty acids in the EWS were used, to subtract the remaining fatty acids from the DM of the EWS as described at Material and Methods. Fatty acids originated from the added xylose were hereby not taken into account.

### HTT pretreatment

#### Composition

The composition of the raw WS (RWS) and the solid fractions of hydrothermally pretreated WS (HTT WS) are compared with the EWS and the solid fractions of pretreated EWS (HTT EWS) (Table 2). The effects of increased temperature in the HTTs are up-concentration of cellulose and lignin in the solid fraction (Table 2).

**Table 2:** Composition of raw wheat straw (RWS) hydrothermal treated wheat straw (HTT WS), ensiled wheat straw (EWS) and hydrothermal treated ensiled wheat straw (HTT EWS) in the solid fraction after HTT (if pretreated)

	**Glucan**	**Xylan**	**Arabinan**	**Lignin**	**Ash**	**Extractives**
	**(w/w % of DM)**					
**RWS**	**40.2 ± 0.2**	**22.3 ± 0.1**	**3.3 ± 0.0**	**18.6 ± 1.1**	**5.2 ± 0.2**	6.3 ± 0.2*
HTT WS 170°C	40.3 ± 2.4	24.8 ± 0.8	2.3 ± 0.1	21.3 ± 0.1	4.8 ± 0.3	
HTT WS 180°C	45.1 ± 1.5	25.2 ± 0.2	2.0 ± 0.0	21.6 ± 0.3	4.0 ± 0.2	
HTT WS 190°C	50.5 ± 0.2	22.4 ± 0.4	1.5 ± 0.2	23.0 ± 0.2	5.0 ± 0.2	
**EWS**	**39.7 ± 0.0**	**24.1 ± 0.4**	**2.6 ± 0.0**	**17.5 ± 1.2**	**3.1 ± 1.1**	**6.9 ± 0.8**
HTT EWS 4w 170°C	40.2 ± 1.0	20.1 ± 1.3	1.3 ± 0.2	23.0 ± 0.4	4.2 ± 0.0	
HTT EWS 4w 180°C	43.2 ± 1.0	18.5 ± 1.2	1.6 ± 0.1	24.5 ± 0.4	4.2 ± 0.3	
HTT EWS 4w 190°C	54.3 ± 0.6	11.8 ± 0.6	0.4 ± 0.0	25.9 ± 0.6	4.0 ± 0.1	

*only ethanol extraction.

Since xylan and arabinan levels in the solid fractions of HTTs are decreasing with increasing HTT temperature, and since levels are lower on EWS, the solubilisation of hemicellulose is concluded to be intensified when the WS is ensiled and the temperature of the HTT pretreatment is increased.

Comparing the glucan content of RWS with that of EWS confirmed that the ensiling effectively preserves the cellulose (Table 2). Likewise, the total amount of fatty acids produced during ensiling (Table 1) is corresponding to the amount of added xylose. Hence, there is no indication of loss of structural carbohydrates during the 4 weeks of ensiling.

#### Mass balance

The glucan content in the pretreated solid fraction plus the small amounts of solubilized glucan were compared to the amount of glucan in the RWS and a total recovery was calculated. The glucan in the EWS was preserved to the same extent as the RWS after HTT and all pretreatments had a recovery above 90% (data not shown).

The pretreatment effect of HTT lies in the mechanism of autohydrolysis, catalyzed by the high temperature steam; here water acts as a weak acid and initiates depolymerization of hemicellulose [28]. During this process acetic acid is released from the O-acetyl groups on the hemicellulose which further enhance the acid hydrolysis [3,29]. The solubilization of hemicellulose, simultaneously with a dislocation of lignin [33] is the reason for inlcreased accessibility to cellulose that facilitates enzymatic attack. Even though the hemicellulose solubilizition is attractive, the hemicellulose carbohydrates still holds potential value in a biorefinery context. The recovery of hemicellulose (xylan and arabinan) is therefore an important factor.

**Figure 1:**
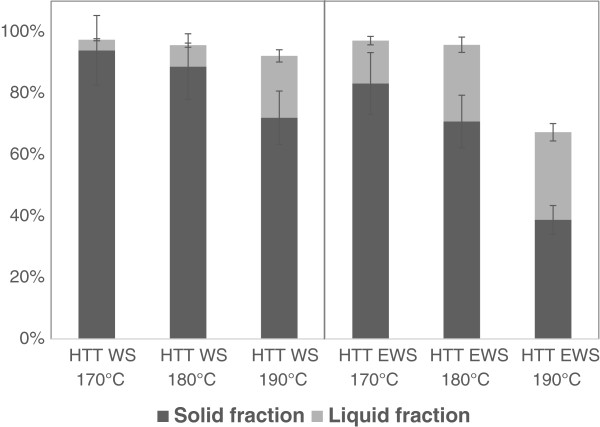
**Recovery of hemicellulose.** Recovery of hemicellulose (xylan and arabinan) in solid fraction (dark) and liquid fraction (light) on HTT treated wheat straw (HTT WS) and on HTT treated ensiled wheat straw (HTT EWS). HTT pretreatment was carried out at 170, 180 and 190°C.

A clear trend was found that temperature increased solubilisation of hemicellulose (Figure 1). For all pretreatments, except HTT EWS 190°C, the hemicellulose was mainly recovered in the solid fraction, and the total recovery for these pretreatments was high (92-97%), while only 64% of the total hemicellulose was recovered from HTT EWS 190°C (Figure 1). The solubilisation of hemicellulose was in general quite low compared to similar studies on hydrothermal pretreatments on WS (e.g. Petersen et al., (2009). [11]). This is most likely due to differences in biomass composition; e.g. Petersen et al. had significantly lower lignin and cellulose content compared to the WS used in this study.

It is clear from the results that ensiling significantly increased the solubilisation of hemicellulose, and the increase with pretreatment temperature was more pronounced (Figure 1). The relative high degradation of hemicellulose for EWS at 190°C indicates that severity of this pretreatment was too high.

**Figure 2:**
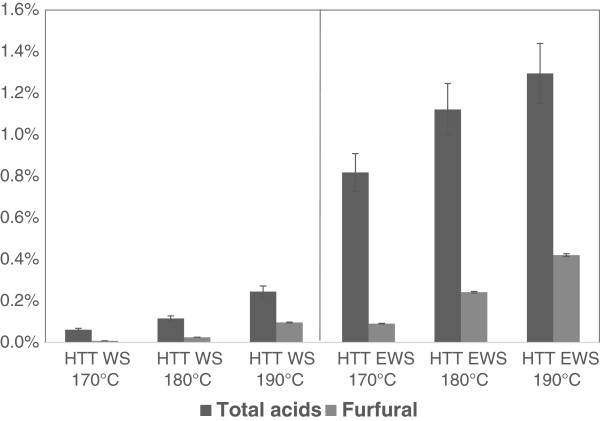
**Organic acids and furfural in liquid fraction after HTT.** Total organic acid (dark) and furfural (light) in (w/w)% of raw material DM. Analyzed in the liquid fractions after HTT treatment of wheat straw (WS) and ensiled wheat straw (EWS).

It is well known that HTT at high temperature and acidic conditions cause degradation of xylose and forms furfural while degradation of glucose mainly forms hydroxymethyl furfural (HMF) and both are potential fermentation inhibitors [10,34]. Accordingly, the increase in hemicellulose degradation with temperature, enforced by the combination with ensiling, was recorded in the measurements of furfural in the hydrolysates (Figure 2). Although the furfural levels were significantly higher in the HTT EWS samples than the HTT WS samples, the maximum concentration did not exceed 0.53 g/L (HTT EWS 190°C), which is far below the critical inhibition levels of 2.0 g/L [35]. HMF concentrations were found not to exceed 0.03 g/L (data not shown) which is likewise much below inhibition levels [35].

For both WS and EWS the concentration of organic acid in the HTT liquid increased with temperature as expected (Figure 2) due to the higher biomass degradation at higher temperature. The HTT EWS liquids had significantly higher concentrations of total organic acids than HTT WS, which was due to both higher biomass degradation but also the organic acid content in the biomass before HTT. The levels on Figure 2 in (w/w)% of DM before HTT is equivalent to between 1.5-1.9 g/L for HTT EWS and 0.1-0.4 g/L for HTT WS. The distribution of organic acids was also different for the WS HTT and EWS HTT. For HTT of WS it was mainly acetic acid and a bit of formic acid, a distribution of 82% and 15% respectively. For the HTT on EWS the distribution was 54% acetic-, 7% formic-, 34% lactic-, and 5% propionic acid (data not shown). The difference in organic acids in the pretreated liquids suggests that the mechanisms during pretreatment of the two different biomasses appear to be different, which is in line with the clear difference in hemicellulose solubilisation (Figure 1). Organic acids can have inhibitory effect in subsequent ethanol fermentation, but for that the concentrations should exceed 10 g/L [35]. On the other hand, it has been shown that inhibitors can serve as very efficient contamination control in large-scale lignocellulosic bioethanol production, preventing growth of especially *Lactobacillus* and thus avoid the need of expensive sterile fermentation equipment [5].

#### Enzymatic hydrolysis

The enzymatic hydrolysis on the pretreated fiber was effectively acting on both cellulose and hemicellulose due to the addition of both cellulase- and hemicellulase blends. The glucose conversion yields in the pretreated solid fraction of the HTT WS increased with temperature especially from 180°C to 190°C where the conversion yield jumped from 45.9 to 71.5% (Table 3). For the HTT EWS the glucose conversion yield ranged from 73.5-78.7% and did not differ significantly due to the standard deviations (Table 2). When addressing the actual release of glucose in (w/w)% of DM in the solid fraction after HTT it were apparent that HTT EWS 190°C gave the highest release of 43.9 (w/w)% (Table 3).

**Table 3:** Glucose conversion after enzymatic hydrolysis of raw wheat straw (RWS), hydrothermal treated wheat straw (HTT WS), ensiled wheat straw (EWS) and of hydrothermal treated ensiled wheat straw (HTT EWS)

	**Released glucose**	**Glucose conversion yield**	**Overall glucose conversion yield**		
			**Liquid fraction**	**Solid fraction**	**Total**
	**In (w/w) % of DM in solid fraction**	**In % of glucose in solid fraction**	**In % of glucose in raw material**	**In % of glucose in raw material**	**In % of glucose in raw material**
**RWS**				19.0 ± 2.6^c^	19.0 ± 2.6^c^
HTT WS 170°C	19.1 ± 0.5^d^	43.0 ± 1.2^b^	0.9 ± 0.0^c^	38.3 ± 1.0^b^	39.1 ± 1.0^b^
HTT WS 180°C	22.8 ± 1.9^d^	45.9 ± 3.9^b^	1.4 ± 0.1^b^	43.0 ± 3.6^b^	44.4 ± 3.6^b^
HTT WS 190°C	39.7 ± 2.9^ab^	71.5 ± 5.1^a^	1.8 ± 0.2^a^	69.3 ± 5.0^a^	71.1 ± 5.0^a^
**EWS**				13.5 ± 0.8^c^	13.5 ± 0.8^c^
HTT EWS 170°C	33.5 ± 2.9^c^	75.7 ± 6.7^a^	0.8 ± 0.1^c^	74.3 ± 6.4^a^	75.1 ± 6.4^a^
HTT EWS 180°C	37.4 ± 1.5^b^	78.7 ± 3.0^a^	1.3 ± 0.1^b^	77.1 ± 3.4^a^	78.5 ± 3.4^a^
HTT EWS 190°C	43.9 ± 2.1^a^	73.5 ± 3.5^a^	1.6 ± 0.1^a^	80.8 ± 3.8^a^	82.3 ± 3.8^a^

Released glucose is expressed as (w/w)% of DM in solid fraction after HTT. Glucose conversion yield is expressed as glucose release in % of glucose in the solid fraction after HTT. Overall glucose yield is the glucose release in the liquid fraction after HTT- and in the solid fraction after enzymatic hydrolysis in % of glucose in the raw wheat straw. The results in each row are grouped according to significance (p = 0.05%), where ‘a’ is significantly higher than ‘b’ and so forth.

The glucose conversion yields after enzymatic hydrolysis were clearly improved by ensiling especially at the lower HTT temperature of 170°C and 180°C, which leads to a significant increase in the overall glucose conversion yields (Table 3). E.g. at the HTT at 180°C the overall glucose conversion yield increased from 44.4% to 78.5% of glucose in raw material when WS was ensiled.The data also showed that ensiling alone was not sufficient as pretreatment, since only 13% of the available glucose in the raw material could be enzymatically converted (Table 3). The low overall glucose conversion yield on WS at the two lower pretreatment temperatures shows that the pretreatment severities were insufficient.

The overall conversion yield of xylose (Table 4) showed the same trend as for glucose (Table 3). However for HTT EWS 190°C the released xylose was significantly lower compared to pretreatments at lower temperatures. This can be explained by the thermal degradation of hemicellulose at higher pretreatment severity. Furthermore, the xylose release of HTT EWS 170°C (17.2 (w/w)%) was similar to HTT WS 190°C (18.0 (w/w)%), corroborating that ensiling facilitated high xylose release at lower pretreatment temperature.

**Table 4:** Xylose conversion after enzymatic hydrolysis of raw wheat straw (RWS), hydrothermal treated wheat straw (HTT WS), ensiled wheat straw (EWS) and of hydrothermal treated ensiled wheat straw (HTT EWS)

	**Released xylose**	**Xylose conversion yield**	**Overall xylose conversion yield**		
			**Liquid fraction**	**Solid fraction**	**Total**
	**In (w/w) % of DM in solid fraction**	**In % of xylose in solid fraction**	**In % of xylose in raw material**	**In % of xylose in raw material**	**In % of xylose in raw material**
**RWS**				14.8 ± 1.7^e^	14.8 ± 1.7^e^
HTT WS 170°C	11.1 ± 0.3^c^	40.0 ± 1.0^d^	3.1 ± 0.0^f^	39.5 ± 0.9^d^	42.6 ± 0.9^d^
HTT WS 180°C	14.6 ± 0.7^b^	51.6 ± 2.6^c^	6.2 ± 0.3^e^	48.6 ± 2.4^c^	54.9 ± 2.4^c^
HTT WS 190°C	18.0 ± 1.6^a^	71.8 ± 6.2^b^	21.1 ± 1.8^c^	55.6 ± 4.8^b^	76.7 ± 4.8^b^
**EWS**				10.5 ± 0.4^e^	10.5 ± 0.4^e^
HTT EWS 170°C	17.2 ± 1.0^a^	76.3 ± 4.6^ab^	14.5 ± 0.0^d^	67.5 ± 4.1^a^	82.0 ± 4.1^ab^
HTT EWS 180°C	16.7 ± 0.8^a^	81.0 ± 5.0^a^	26.7 ± 2.3^b^	61.1 ± 3.1^a^	87.8 ± 4.9^a^
HTT EWS 190°C	11.7 ± 0.7^c^	88.2 ± 5.5^a^	30.6 ± 0.0^a^	37.9 ± 2.3^d^	68.5 ± 2.3^d^

Released xylose is expressed as (w/w)% of DM in solid fraction after HTT. Xylose conversion yield is expressed as xylose release in % of xylose in the solid fraction after HTT. Overall xylose yield is the xylose release in the liquid fraction after HTT- and in the solid fraction after enzymatic hydrolysis in % of xylose in the raw wheat straw. The results in each row are grouped according to significance (p = 0.05%), where ‘a’ is significantly higher than ‘b’ and so forth.

The positive effect of ensiling WS prior to HTT can be quantified by comparing the yields over the same pretreatment temperature. At 170°C and 180°C ensiling improves the total yield. Comparing the released glucose and xylose (Table 3 and Table 4) from HTT WS with HTT EWS it can be concluded that we gain substantial more released sugar than the 7% xylose spent facilitating the ensiling process. However, at 190°C this positive sugar balances is not observable due to xylose degradation.

The literature points at two main reasons for the improved sugar release of combining ensiling and HTT. First, the improved sugar release is connected to the natural long term impregnation of organic acids on the biomass where the lignocellulosic structure is loosened by weak acid hydrolysis accomplished by organic acids [6]. Due to the addition of xylose as substrate for ensiling, it could not be concluded to which extent hemicellulose was solubilized, but the combined results suggests very little solubilisation. Since this study did not look at the duration of the ensiling or included pretreatment of WS merely soaked in organic acids as a control, it cannot be unequivocally concluded that the improvement of HTT on EWS was directly due to the long term ensiling alone. Monavari *et al*. [36] did a study on impregnation with lactic acid on bagasse prior to steam explosion and found a significant difference between long term impregnation (4 weeks) and merely soaking, favoring the impregnation, proving that this is in fact a factor. Nonetheless, soaking of the dry wheat straw to a DM of 35%, do cause swelling of the cell wall, which is most likely improving the effect of pretreatment.

The second main effect of ensiling prior to HTT is the lowering of pH which causes higher severity, i.e. the action of the produced organic acids within the HTT pretreatment. Especially acetic acid, but also lactic acid has been shown to catalyze the autohydrolysis and improve the process as it was found by Xu *et al*. [27]. Recently it has been shown that addition of 0.04 g (g DM)^-1^ acetic acid to HTT of wheat straw increased glucose yield at both 190°C and 195°C, however not at 200°C, thus the effect of acetic acid was more significant at lower temperatures [37]. Results from the present study also determine that improvement by acid catalyzed autohydrolysis increases at decreasing pretreatment temperature. Furthermore, due to the large effect of ensiling at lower HTT temperatures i.e. 170-180°C, it would be interesting to test even lower HTT temperatures than 170°C in future studies.

## Conclusion

Ensiling prior to hydrothermal treatment was shown to significantly increase the effect of the pretreatment, especially at 170°C, and 180°C. An effective ensiling of wheat straw was accomplished with the presented method in which both glucan and xylan was effectively preserved, and where the DM loss during ensiling was under 0.5%. Ensiled wheat straw hydrothermally treated at 180°C gave the highest overall conversion yield regarding both glucan and xylan, 73.6% and 83.5% respectively, but even pretreatment of ensiled wheat straw at 170°C provided satisfying results, 70.4% and 77.4% for glucan and xylan respectively. In both cases, more xylose was gained after the enzymatic hydrolysis than was used in the production of the wheat straw silage. The findings potentially enable a considerable decrease in the necessary process temperature in hydrothermal treatments of wheat straw, thereby having a positive effect on large scale pretreatment costs.

## Materials and methods

### Raw material

Wheat straw (*Triticum aestivum* L.) was supplied by DONG Energy (Skærbæk, Denmark). The straw was chopped to approximately 10 cm pieces and stored at ambient temperature. Dry matter content of the stored WS was 90%.

#### The process

Combined ensiling and HTT pretreatment was tested against conversion of glucose and xylose after subsequent enzymatic hydrolysis. The combined pretreatment (HTT EWS) were compared to the conversion in raw wheat straw (RWS), ensiled wheat straw (EWS) and sole HTT pretreated wheat straw (HTT WS).

#### Ensiling

Ensiling was carried out on chopped WS (10 cm) adjusted to 35% final DM content. Due to the low free sugar content of WS, 7 g xylose per 100 g DM was added as determined to be optimal by Yang *et al*. [14]. Each batch of ensiling contained 1.5 kg DM WS. The ensiling was carried out using a vacuum based plastic bag system [38] and a Variovac EK10 vacuum packaging machine (Variovac Nordic A/S, DK-7100 Vejle, Denmark).

The commercially available inoculum LACTISIL CCM (Chr. Hansen, Hørsholm, Denmark) which consists of freeze dried pure heterofermentative *Lactobacillus buchneri* was applied. A suspension of 0.2 g L^-1^ water was prepared and added in the amount of 40 mL kg^-1^ WS to reach an initial inoculum size of 8 mg kg^-1^.

The plastic bags were opened after 4 weeks. Weight loss was measured for calculation of DM loss. After ensiling, 1 kg DM of the ensiled WS was pretreated hydrothermally.

#### Hydrothermal pretreatment

Hydrothermal pretreatments (HTT) were carried out in the “Mini IBUS” equipment (Technical University of Denmark, Risø campus). 1 kg DM (corrected for volatile fatty acid) of the EWS was treated at different temperatures (170, 180 and 190°C) for 10 min. In order to verify the reproducibility of HTT, the EWS pretreated at 180°C were done in triplicate. After HTT the pretreatment reactor was cooled to below 70°C thereby avoiding evaporation of acids, and the material was separated by pressing. Each solid fiber fraction and each liquid fraction were analyzed separately. The solid fraction was kept in the freezer and used to evaluate the process efficiency by enzymatic hydrolysis.

#### Enzymatic hydrolysis

The enzymatic convertibility assay based on commercial CellicCTec2 (blend of cellulases) and CellicHTec2 (blend of hemicellulases) (Novozymes A/S, Denmark) was used to determine the efficiency of the pretreatment process. Enzymatic conversion of pretreated solids was performed at 5% DM content in a total volume of 25 mL using 50 mM citrate buffer (pH 5) and 0.25 mL sodium azide (2%) at 50°C shaken at 150 rpm for 72 h. Applied enzyme loadings were 15 FPU g^−1^ DM solids of CellicCTec2 supplemented with xylanase CellicHTec2 (90:10 ratio based on protein loading for all assays). The enzymatic hydrolysis was performed in triplicates and enzyme blanks were included. Samples were analyzed for carbohydrates on HPLC. Cellulose convertibility was calculated as the converted cellulose divided by the original cellulose content.

#### Chemical analysis

Raw wheat straw (RWS), ensiled wheat straw (EWS), hydrothermally pretreated wheat straw (HTT WS) and hydrothermally pretreated ensiled wheat straw (HTT EWS) were analyzed for chemical composition by methods based on standard laboratory analytical procedures developed by National Renewable Energy Laboratory (NREL), US [39]. Deviations from these standard procedures are stated in the following sections. The analysis of the solid fiber fraction included ash content determination, water extraction, ethanol extraction and strong acid hydrolysis for structural carbohydrates and lignin. The liquid fraction of the HTT was analyzed by weak acid hydrolysis.

#### DM determination

DM was determined using a standard method [39]. The contribution of fatty acids produced during ensiling was subtracted from the DM, since the acids originated from the added xylose, which likewise were not included in the original DM content of WS. Huida *et al*. [40] determined volatilization coefficients describing to which extent different fatty acids were evaporating during determination of DM at specific pH. These volatilization coefficients were used to determine how much of the different acids that were left after DM determination of EWS in order to correct for this amount. Fatty acids in RWS and solid fraction of HTTs EWS were negligible, thus no correction of DM were needed in these cases.

#### Analytical method

Concentrations of carbohydrates (d-glucose, d-xylose, l-arabinose), organic acids (lactic-, formic-, acetic-, propionic, and butyric acid) were quantified by HPLC using a Biorad HPX-87H column (Hercules, CA; USA), RI detector, 63°C and 4 mM H_2_SO_4_ as eluent, at flow rate of 0.6 ml min^-1^.

#### Water extraction

0.3-0.4 g DM biomass from freshly disrupted silage bags was extracted in 10 ml MilliQ H_2_O with 10 μl of the antibiotic ampicillin (10 mg/ml solution) to prevent microbial activity during extraction. The extraction samples were shaken for 2 hours at 25°C and 150 rpm. Extracts were analyzed for sugars, acids by HPLC as described above. Acids produced from additional xylose used for initiating ensiling process, were taken into account.

#### Weak acid hydrolysis of hydrolysates

The liquid fraction of HTT was further analyzed by weak acid hydrolysis to quantify the content of soluble oligomer carbohydrates. 10 ml HTT liquid fraction were autoclaved for 10 minutes at 121°C with 4 w/w % H_2_SO_4_. Derived sugars were analyzed by HPLC as described above.

#### Ethanol extraction

Lipophilic extraction was carried out by Soxhlet extraction in a reflux condenser for six hours with 99 w/w% ethanol on water extracted samples of EWS. The amount of ethanol extractives, including volatiles, was defined as the mass of material lost through extraction.

#### Determination of structural carbohydrates and lignin

Strong acid hydrolysis was used to measure the carbohydrate and lignin content of the extracted bio residue, based on the NREL standard laboratory analytical procedure [32].

#### Statistical evaluation

One-way analyses of variances (one-way ANOVA): 95% confidence intervals were compared as Tukey–Kramer intervals calculated from pooled standard deviations (Minitab Statistical Software, Addison-Wesley, Reading, MA).

## Abbreviations

HTT: Hydrothermal treatment; WS: Wheat straw; EWS: Ensiled wheat straw; RWS: Raw wheat straw; DM: Dry matter; HPLC: High-performance liquid chromatography.

## Competing interests

The authors declare that they have no competing interests.

## Authors’ contributions

MA participated in the experimental design, carried out the ensiling treatment, contributed to acquisition of data and drafted the manuscript. STT participated in the planning and executing the laboratory work, contributed to acquisition of data and reviewing the manuscript. ZK participated in the experimental design, contributed to acquisition of data and review the manuscript. AM contributed to acquisition of data, performed statistical analyzis and review the manuscript. MA and STT contributed equally to this work. All authors read and approved the final manuscript.
